# Diagnostic stewardship for blood cultures in the pediatric intensive care unit: lessons in implementation from the BrighT STAR Collaborative

**DOI:** 10.1017/ash.2024.416

**Published:** 2024-09-25

**Authors:** Charlotte Z. Woods-Hill, Danielle W. Koontz, Anping Xie, Elizabeth A. Colantuoni, Anna Sick-Samuels, Marlene R. Miller, Abigail Arthur, Anushree Aneja, Urmi Kumar, Aaron M. Milstone, for the Brigh T STAR authorship group

**Affiliations:** 1 Division of Critical Care Medicine, The Children’s Hospital of Philadelphia, University of Pennsylvania Perelman School of Medicine, Philadelphia, PA, USA; 2 Division of Infectious Diseases, Department of Pediatrics, Johns Hopkins University School of Medicine, Baltimore, MD, USA; 3 Department of Anesthesiology and Critical Care Medicine, Johns Hopkins University School of Medicine, Baltimore, MD, USA; 4 Department of Biostatistics, Bloomberg School of Public Health, Johns Hopkins University, Baltimore, MD, USA; 5 Rainbow Babies and Children’s Hospital, Cleveland, OH, USA; 6 Case Western Reserve University School of Medicine, Cleveland, OH, USA

## Abstract

**Objective:**

BrighT STAR was a diagnostic stewardship collaborative of 14 pediatric intensive care units (PICUs) across the United States designed to standardize and reduce unnecessary blood cultures and study the impact on patient outcomes and broad-spectrum antibiotic use. We now examine the implementation process in detail to understand how sites facilitated this diagnostic stewardship program in their PICUs.

**Design:**

A multi-center electronic survey of the 14 BrighT STAR sites, based on qualitative data about the implementation process collected during the primary phase of BrighT STAR.

**Setting:**

14 PICUs enrolled in BrighT STAR.

**Participants:**

Site leads at each enrolled site.

**Methods:**

An electronic survey guided by implementation science literature and based on data collected during BrighT STAR was administered to all 14 sites after completion of the primary phase of the collaborative.

**Results:**

10 specific tasks appear critical to implementing blood culture diagnostic stewardship, with variability in site-level strategies employed to accomplish those tasks. Sites rated certain tasks and strategies as highly important. Strategies used in top-performing sites were distinct from those used in lower-performing sites. Certain strategies may link to drivers of culture overuse and represent key targets for changing clinician behavior.

**Conclusions:**

BrighT STAR offers important insights into the tasks and strategies used to facilitate successful diagnostic stewardship in the PICU. More work is needed to compare specific strategies and optimize stewardship outcomes in this complex environment.

**Clinical trial registry information:**

Blood Culture Improvement Guidelines and Diagnostic Stewardship for Antibiotic Reduction in Critically Ill Children (Bright STAR). NCT03441126. https://www.clinicaltrials.gov/study/NCT03441126?term=Bright%20STAR&aggFilters=status:com&checkSpell=false&rank=1

## Introduction

Medical overuse—care in which net benefits do not exceed net harms—comprises up to one-third of United States healthcare spending and is associated with excess cost, worse patient outcomes, and death.^1–6^ Overtesting is a type of overuse in which non-informative screening tests are performed on asymptomatic patients, or symptomatic patients undergo more testing than is necessary.^7^ The consequences of overtesting are significant: false positive results, needless follow-up studies and treatments, patient anxiety, and hundreds of millions of dollars per year.^7^

A high-impact approach to combat overtesting is diagnostic stewardship—optimizing the use of diagnostic tests to improve treatment decisions.^1^ Diagnostic stewardship programs have successfully reduced urine cultures, respiratory tract cultures, and *Clostridioides difficile* (*C. difficile*) testing in various settings.^8–12^ As bacterial cultures are often coupled with starting empiric antibiotics, stewardship of microbiology testing is emerging as an effective approach to reduce antibiotic overuse.^13^

Currently, limited literature exists to guide the process of implementing diagnostic stewardship in healthcare settings.^14^ A recent collaborative called BrighT STAR (Testing STewardship for Antibiotic Reduction) provides an opportunity to explore successful implementation of diagnostic stewardship. BrighT STAR focused on the pediatric intensive care unit (PICU) environment and introduced blood culture diagnostic stewardship into 14 PICUs from 2017 to 2021.^15^ Participating sites were geographically diverse and were a mix of cardiac (40%) and non-cardiac units (60%), but the majority provided at least tertiary-level services with surgical, oncological, and stem cell transplant care, as well as advanced modes of ventilation and extracorporeal membrane oxygenation, and all were academic with critical care fellowship programs.^15^ Sites were selected with a goal of diversity in size and geographic distribution, ability to obtain the required data metrics, and commitment to participation from both a pediatric intensivist and a pediatric infectious disease physician. All sites had an existing antimicrobial stewardship program. BrighT STAR’s results were compelling: a 33% reduction in blood culture rates, a 13% reduction in broad-spectrum antibiotic use, no significant change in safety balancing measures, and sustainability of its primary outcome.^15,16^ Subsequent qualitative work found seven distinct determinants and three specific types of cognitive bias that appear to be key drivers of blood culture decisions by PICU clinicians.^17^

On a programmatic level, BrighT STAR was anchored by principles participatory ergonomics and quality improvement.^15,18^ Participatory ergonomics is the application of human factors and ergonomics to work system design and emphasizes the involvement of the people who actually perform the task of interest, as they have sufficient knowledge and power to influence processes and outcomes to achieve the desired goals.^18^ Sites were guided through core steps and demonstrated variations in both the clinical interventions and the implementation process.^18^ Our objectives now were to describe the BrighT STAR site-level implementation processes, characterize specific strategies for changing blood culture practices, explore whether these strategies link to the determinants of culture overuse, and correlate the strategies associated with successful implementation of a blood culture diagnostic stewardship program in the PICU environment.

## Methods

### Brief background: the BrighT STAR Collaborative core steps, tasks, and site-level strategies

**Figure 1.**
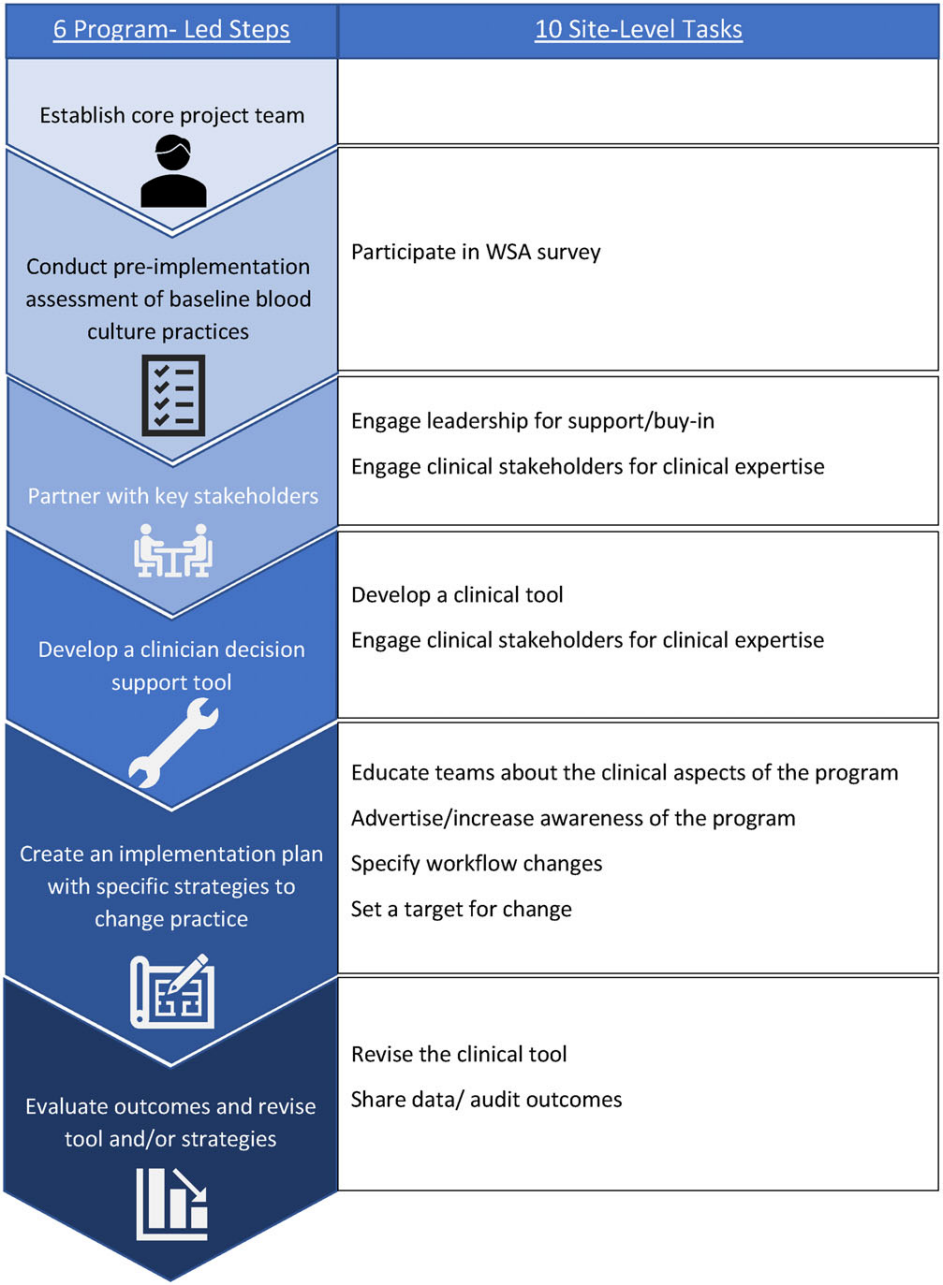
Six steps and ten tasks for diagnostic stewardship during the BrighT STAR Collaborative.

Overall, we sought to maintain fidelity to core elements of the diagnostic stewardship program while allowing adaption of some of those elements to better fit with local unit culture and local stakeholder priorities.^15,18^ All 14 sites in BrighT STAR completed the same series of six steps guided by the study team: establish a core project team of pediatric critical care and pediatric infectious disease physicians (with additional team members at sites’ discretion), participate in a pre-implementation assessment of current blood culture practices, partner with key institutional stakeholders, develop a clinical decision support tool, create an implementation plan with specific strategies to change blood culture practices, and then monitor outcomes and revise the tool and/or strategies as needed.^15^ Although the BrighT STAR team guided each site through these shared steps, execution of those steps involved distinct *tasks* and specific *strategies* to accomplish those tasks. We are defining “tasks” as actions taken by all or most sites to address or complete the 6 core BrighT STAR steps, while “site-level strategies” (SLS) are the specific, and variable, ways in which tasks were accomplished at the different sites (Figure 1 and Table 1). For example, if the step was “partner with key stakeholders,” the associated task was “engage leadership for support/buy-in,” and one identified strategy would be “give a formal presentation.” During the collaborative, we asked project champions at each site to document their activities in a site diary and share them with the study team at regular intervals.

**Table 1.** Summary of BrighT STAR Collaborative process—program-led steps, site-level tasks, and site-level strategies

6 Program-led steps	10 Site-level tasks	Site-level strategies	Number of sites using the strategy
1. Establish core project team of ID/PICU/+other (trainee, nurse, QI expert)	N/A	N/A	N/A
2. Conduct pre-implementation assessment of baseline blood culture practices	I. Participate in WSA survey	Use WSA survey results to create clinical tool and develop implementation plan	14/14 (100%)
3. Partner with key stakeholders	II. Engage leadership for support/buy-in	Formal presentation, more than once	14/14 (100%)
		Informal discussion, more than once	14/14 (100%)
		Email discussion	14/14 (100%)
	III. Engage clinical stakeholders for clinical expertise*	Formal presentation, more than once	14/14 (100%)
		Informal discussion, more than once	14/14 (100%)
		Email discussion	14/14 (100%)
4. Develop a clinician decision support tool	III. Engage clinical stakeholders for clinical expertise*	Formal presentation, more than once	14/14 (100%)
		Informal discussion, more than once	14/14 (100%)
		Email discussion	14/14 (100%)
	IV. Develop a clinician decision support tool	Paper checklist, of any type	12/14 (86%)
		Paper checklist, required completion or collected for review	7/14 (50%)
		Paper checklist, but completion not required/not collected for review	6/14 (43%)
		EMR/electronic tool	2/14 (14%)
5. Create an implementation plan with specific strategies to change practice	V. Educate teams about the clinical aspects of the program	Formal presentation, once/more than once	14/14 (100%)
		Informal discussion, once/more than once	14/14 (100%)
		Flyers posted around the unit/workspaces	13/14 (93%)
		Emails to sites’ clinicians	14/14 (100%)
		Online module	1/14 (7%)
	VI. Advertisement/awareness of the program	Formal session	14/14 (100%)
		Informal discussion	14/14 (100%)
		Visual reminders/flyers	12/14 (86%)
		Email	13/14 (93%)
	VII. Workflow changes	Integrate physical examination prior to culture decision	13/14 (93%)
		Attempt peripheral venipuncture	11/14 (79%)
		Change sample collection order	11/14 (79%)
		Create new order set	1/14 (7%)
		Create a new team	1/14 (7%)
		Integrate into sepsis huddle	3/14 (21%)
	VIII. Set a target for change	For example, “reduce total culture rate by 20% within 6 months”	9/14 (64%)
**Launch Program**			
6. Evaluate outcomes and revise tool and/or strategies	IX. Revise the clinical tool	Paper, One time	7/14 (50%)
		Paper, More than once	7/14 (50%)
		Online tool edits, one time	2/14 (14)
		Online tool edits, more than once	0/14 (0%)
		No edits at all	7/14 (50%)
	X. Share data/ audit outcomes	Share compliance data internally	14/14 (100%)
		Share site performance data internally	11/14 (79%)
		Share OTHER sites’ performance data within your site	7/14 (50%)

Note. *Site-level Task III is part of both Program-led Step 3 and Step 4.

### Data collection

After completion of the collaborative, the study team created an electronic survey to capture additional data on tasks and SLS used in each site (Qualtrics, Provo, UT). The survey was based both on implementation science literature focusing on how to classify and analyze strategies used to change behavior, and on the qualitative data gathered from the site diaries used during the primary phase of the project.^18^ The survey was designed to evaluate tasks and SLS in multiple domains.^19^ The survey aims were to document the tasks and strategies used by each site and then assess the site team’s perceptions around executed tasks and strategies regarding: (*i*) importance to facilitate blood culture practice change within their site, (*ii*) the degree of personal effort/time required to execute, and (*iii*) the degree of financial or external resources required to execute the task/strategy. The survey focused on the tasks/SLS that could be variable across the sites. The survey questions were organized into three categories corresponding to Leeman et al.’s framework for strategy analysis: dissemination strategies (awareness, attitude, knowledge, and intention to adopt a specific practice), implementation process strategies (how well teams execute activities required to select, adapt, and integrate the new practice generally), and integration strategies (factors that facilitate or impede optimal integration of a specific practice into a specific setting) (Supplement 1).^19^

The survey was sent by email to the BrighT STAR primary site leads for all 14 sites, with inclusion of additional key team members at the discretion of the site. Individual survey responses were anonymous but identified by site. Johns Hopkins was the coordinating center, and The Johns Hopkins Institutional University Review Board deemed this exempt research.

### Analysis

Survey data were analyzed both quantitatively and qualitatively. First, to document which tasks/SLS were used in different sites to accomplish the tasks and steps related to BrighT STAR, completion of a task or SLS was defined as a site answering “yes” on questions that asked if a particular task or SLS was or was not done (to account for potential variability in individual recollections and inconsistent responses within a site). Second, to assess sites’ perception of the importance of each task/SLS, aggregate responses from the 14 sites were used to classify each tasks/strategy as “highly important” if the majority (≥50% of respondents) rated it as extremely or very important, and scores below this combined into a “low-moderately” important category. Similarly, for resources and effort, “high” resources or effort were defined as >50% of respondents rating a task or strategy as “extremely” or “very” resource-intensive or effortful, and scores below this were considered “low-moderate” resources or effort. To highlight if any of the tasks/SLS were associated with site performance (measured by % reduction of blood cultures), the top 3 performing sites and the bottom 3 performing sites (ranked by relative rate reduction in blood cultures pre- versus post-implementation in our BrighT STAR collaborative) were grouped and compared to assess if there was association of strategy used or rating with a site’s performance in reducing blood culture rates. Finally, in a qualitative exploratory analysis, SLS used in the sites in relationship to the determinants uncovered in our earlier interviews were examined to discover if any strategy was linked mechanistically to any particular determinant.^16^

## Results

All sites (14/14) provided data to the study team throughout the project period including their site diary, copies of the clinical decision tools developed at each site, and information about the workflow changes or processes implemented to facilitate the blood culture program. All sites (14/14) completed the survey, with 31 individual responses and median of 2.2 responses per site.

### Tasks and site-level strategies identified

Ten specific tasks that most of the 14 sites addressed during the course of implementing blood culture stewardship were identified: engage leadership for support/buy-in, engage clinical stakeholders for devising the new clinical approach to blood cultures, complete the Work System Assessment to assess baseline practices and context, educate PICU clinicians about the clinical elements of the program, advertise the program within the PICU, set a target for change, develop a specific clinical decision support tool, revise that tool, share data with the study team, and share data within a site or institution (Table 1 and Figure 1).

Key similarities and differences in the specific SLS employed to complete these 10 tasks are summarized in Table 1. For certain tasks, the specific strategies used by the sites to accomplish that task were very similar across the collaborative: 100% of sites used formal presentations, informal discussions, and emails to engage leadership, engage clinical stakeholders, educate PICU clinicians about the clinical components of BrighT STAR, and advertise the program. Most sites (93% and 86%, respectively) used visual reminders/flyers to educate and increase awareness of the program. Most sites (93%) specifically emphasized the completion of a physical examination prior to decision about obtaining a blood culture as a workflow change strategy. For other tasks, the SLS used were more variable: for example, 9/14 sites set a specific target for change at the start of the program (such as “reduce cultures by 15%” or “decrease use of surveillance cultures on ECMO patients”); 3/14 attempted to integrate the blood culture program into existing sepsis huddles, and 1/14 set up a new team to collect blood cultures (Table 1).

Most sites (86%) created a paper checklist as the decision support tool, with notable variability in how that checklist was used. In half of sites, that checklist was either required to be completed by a clinician before a blood culture was ordered or was collected and reviewed by a member of the site team as an assessment of compliance with the new stewardship approach; while in 6/14 sites, the checklist was offered as guidance but completion or review of the checklist was not performed. Only 2/14 sites utilized electronic medical record infrastructure to support their stewardship program (EMR decision support and a new order set).

### Importance of specific tasks and site-level strategies

Sites rated their perception of the importance of both tasks and the specific SLS used to complete those tasks. Four tasks and seven SLS were rated as “highly important” by the respondents in aggregate (Table 2 and Table 3). There was some discordance among sites regarding perception of the effort or resource burden required to complete a task, even if a task was universally rated as “important” (eg, 6 sites rated “clinical stakeholder engagement” as requiring a high level of effort or resources, while the other 8 sites rated that task as requiring only moderate or low effort/resources) (Table 2). Other tasks, such as “leadership buy-in” were deemed to need low or moderate effort/resources across all 14 sites. Similar variability was noted for the SLS included in Table 2, with a mix of low/moderate and high effort/resources seen across the sites.

**Table 2.**
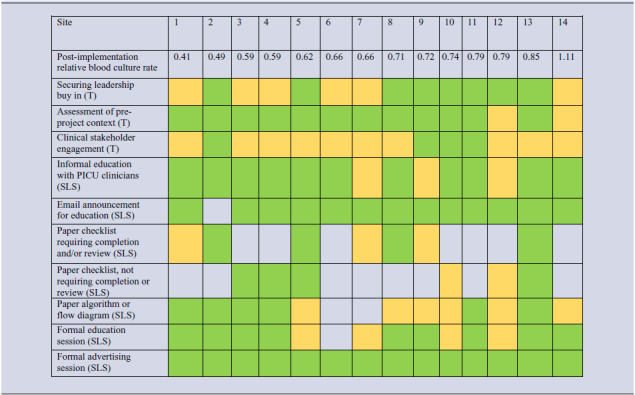
Tasks (T) and site-level strategies (SLS) rated as “highly” important, defined as >50% of survey responses rating a strategy as “extremely” or “very” important. Color indicates that a site did a task or used a strategy, defined as any “yes” response from a site team member. Blank space/white indicates that a site did not do the task nor use the strategy. Yellow indicates that a site rated that task or strategy as extreme or high amount of either resources or efforts required. Green indicates that a site rated that task or strategy as moderate, low, or no resources or effort required. Sites (columns) are ordered by the relative reduction in blood culture rate achieved during the BrighT STAR collaborative

Green: High importance, low or moderate resource intensity/effort.

Yellow: High importance, high resource intensity/effort.

White: not implemented at site.

**Table 3.** Matrix of task (T) and site-level strategy (SLS) importance versus resource intensity or effort level required, as rated by the BrighT STAR sites. High importance was defined as >50% of total survey responses across all 14 sites rating a task/strategy as “extremely” or “very” important; scores below this were defined as low-moderate importance. High resources or effort were defined as >50% of total survey responses across all 14 sites rating a task/strategy as “extremely” or “very” resource-intensive or effortful; scores below this were defined as low-moderate effort/resources

	Low-moderate importance	High importance
Low-moderate resources/effort	Screen saver/posting flyer (SLS)	Securing leadership buy in (T) Informal education with PICU clinicians (SLS) Email announcement for education (SLS) Paper algorithm or flow diagram (SLS)
High resources/ effort	None	Assessment of pre-project context with Work System Assessment (T) Clinical stakeholder engagement (T) Paper checklist requiring completion and/or review (SLS) Paper checklist, not requiring completion or review (SLS) Formal education session (SLS) Formal advertising session (SLS) Auditing compliance to the clinical tool (T)

Note. *Did not include strategies used by very few number of sites: EMR-based tool, online module, sepsis huddle integration.

### Strategies used in high-performing sites

Several observations are noteworthy when comparing SLS used by the three sites with the highest and lowest relative reduction in blood culture use during the BrighT STAR collaborative (Table 2). In the top three performers, two sites set a target for change, used a checklist that required completion and/or review, and shared data about site-specific project results and collaborative-wide results with non-project members of their PICU or institution (along with members of their site team). In contrast, in the bottom three performers, only one site set a target, used a checklist, or shared results data beyond what was required by the BrighT STAR study team.

### Connection between tasks or strategies and determinants of culture overuse

**Figure 2.**
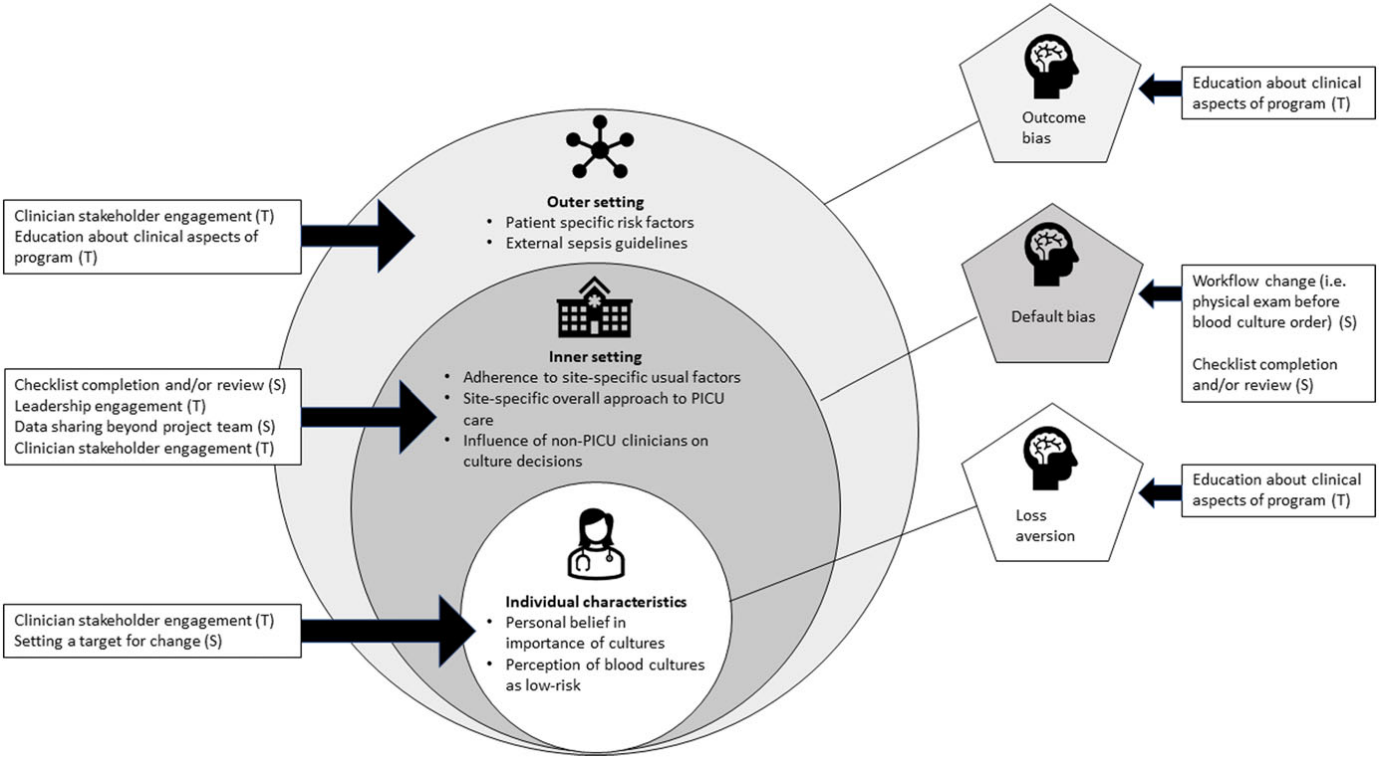
Mapping determinants of blood culture use with diagnostic stewardship TASKS (T) and specific practice change STRATEGIES (S) used by BrighT STAR sites.

Finally, we explored for any conceptual connection between the tasks and/or SLS used by sites and the determinants of overuse identified in our earlier qualitative work (Figure 2).^17^ For example, securing leadership buy-in through intentional engagement may address the belief that blood cultures are a low-risk test not needing to be reduced, by showing that highly influential figures support the practice change. Using a checklist that requires completion or review and integrating a physical examination before a blood culture order may mechanistically target both default bias and site-specific approach to care, by creating an intentional pause before a clinician practices in their usual or default way. Sharing data widely may target the influence of non-PICU clinicians on blood culture practices by including outside stakeholders in project progress.

## Discussion

During the BrighT STAR collaborative, 14 PICUs implemented a diagnostic stewardship program focusing on standardizing and improving blood culture practices using a common series of steps guided by the study team. Examining the implementation process in more detail has now yielded important insights about how to facilitate diagnostic stewardship in the PICU environment.

First, these findings identified ten discrete tasks that a site likely needs to address to implement blood culture stewardship in the PICU. Many of these tasks are basic steps that any quality improvement project would likely tackle, but this is the first effort to synthesize and illustrate key actions for facilitating stewardship of this particular clinical test in this specific setting. These 10 tasks offer specific guidance about how to approach the multidisciplinary and complex nature this particular topic. For example, “engaging stakeholders” is fundamental to any QI work, but for blood culture stewardship, the critical stakeholders include PICU and infectious diseases physicians (universally across all sites), front-line ordering clinicians (the types of which may vary depending on institutional roles), nursing, and non-PICU clinical groups, such as oncology or surgery.

Second, while we did not a priori design these ten tasks using a specific implementation science tool, examination now demonstrates that the tasks align well with the Clinical Sustainability Assessment Tool (CSAT).^20^ The CSAT is an instrument designed to facilitate understanding of contextual factors that enable sustainable clinical practice changes, and has 7 domains: engaged staff and leadership, engaged stakeholders, monitoring and evaluation, organizational context and capacity, workflow integration, planning and implementation, and outcomes and effectiveness. Our ten tasks overlap to a strong degree with these seven domains. CSAT is emerging as an important tool that can guide work such as ours to lead to long-term, meaningful change in clinical practices. Subsequent BrighT STAR collaborative work to improve respiratory culture stewardship is currently using the 7 CSAT domains to guide its diagnostic stewardship efforts, and those results are anticipated in the near future.

Third, it appears that paper checklists were the primary type of clinical decision tool used in most sites; with half requiring completion or collection for review. Only two sites tried to use any EMR-associated tool. There are several possible reasons for this surprisingly low-tech approach: (*i*) blood culture stewardship was novel and relatively unexplored in our sites, and paper tools are easy to iteratively revise during the course of a project; (*ii*) blood culture stewardship is predicated on when not to send a test, unlike many other clinical pathways or decision support tools, which are designed to tell a clinician what to do or when to do something. In addition, deciding to defer a blood culture in a PICU patient is an inherently complex decision that requires clinician judgment and a thorough understanding of specific patient’s clinical status. Designing something like a “best practice alert” may be challenging when the goal is to avoid unnecessary testing and when there is no objective or definitive rule in place (eg, unlike an alert for avoiding blood transfusions when there is a laboratory value and evidenced-based practice guidelines on thresholds for transfusion).

Next, our data gives some preliminary insights into the relative importance versus resources or effort required for specific SLS used (Tables 2 and 3). Understanding which strategies are likely to be high-yield versus low-yield, and how much time or effort will be required to use those strategies, informs program implementation in new sites. These results suggest that securing leadership buy-in, informal education with PICU clinicians, and email announcements are the “low hanging fruit” for beginning blood culture stewardship, while several strategies (such as assessment of pre-project context, clinical stakeholder engagement, and formal education/advertising sessions) may require more effort but are likely important tasks to facilitate project success.

Finally, while limited by small sample size, the data suggest that some elements of strategy did differ between high and low performing sites (Table 2). Some of the actions of the top 3 performers, like setting a target for change, using a checklist that required completion and/or review, and sharing data about project results with non-project team members may have served important functions such as changing reflexive clinician behavior and enhancing project momentum by audit/feedback of performance.^21^ These strategies may also act to target specific determinants of overuse, but more work is needed to fully understand the mechanisms of behavior change that may facilitate successful diagnostic stewardship. Also notable was the finding that lower-performing sites rated more strategies as requiring higher levels of resources or effort (Table 2). There may be a link between finding it more challenging to complete core, important tasks such as clinical stakeholder engagement and formal education sessions and ultimate ability to successfully change practice. Future diagnostic stewardship efforts in new PICUs should proactively address specific challenges with the important tasks identified here to optimize success.

Our study has important limitations. In additional to our small sample size and limited generalizability, recall bias inherent in survey methodology may have affected some of the respondents’ answers. Our survey instrument was also developed specifically for this work and consequently has limited formal validation. We also acknowledge that each site had an active antimicrobial stewardship team in place during BrighT STAR, but no formal process exists to evaluate if these programs had high variability in their resources or program infrastructure, which may have affected how they approached this diagnostic stewardship work.

Although complex and likely strongly influenced by local context and culture, evaluation of the 14-site BrighT STAR collaborative highlighted core tasks and specific strategies for blood culture diagnostic stewardship. Variability in strategies and in how much effort or resources may be required for each strategy highlights opportunities to fine-tune the implementation approach in additional institutions. Aligning stewardship efforts to address core domains described in the CSAT tool may optimize outcomes. Important future work includes better understanding if and how any of these strategies may link mechanistically to determinants of blood culture overuse, and if select strategies lead to practice change most effectively.

## Supporting information

Woods-Hill et al. supplementary material 1 Woods-Hill et al. supplementary material

Woods-Hill et al. supplementary material 2 Woods-Hill et al. supplementary material

## Supplementary material

The supplementary material for this article can be found at https://doi.org/10.1017/ash.2024.416.

## Research transparency and reproducibility

BrighT STAR sites have a data use agreement that does not cover sharing sites’ data beyond the scope of that agreement, therefore we are unable to publicly share data.
