# Supplementary material for: Diagnostic stewardship for blood cultures in the pediatric intensive care unit: lessons in implementation from the BrighT STAR Collaborative
Source: Antimicrob Steward Healthc Epidemiol. 2024 Sep 25;4(1):e148. doi: 10.1017/ash.2024.416 (PMC11428017; doi:10.1017/ash.2024.416)
Supplement: Woods-Hill et al. supplementary material 1 — Woods-Hill et al. supplementary material [file S2732494X24004169sup001.docx]

Bright STAR Blood Strategy Survey

The intent of this survey is to gather information about what strategies you used in your site during the Bright STAR blood culture quality improvement project. By “strategy,” we mean, activities that you/your site used in order to optimize blood culture practices within your PICU.

This survey should take approximately 20 minutes to complete. Completing this survey is strictly voluntary and your responses are confidential. Your completion of this survey will serve as your consent to be in this research study.  All results will be reported in aggregate form so that no one person can be identified. Your input is invaluable to the success of this project.

Thank you from the Bright STAR team.  For questions, please email Dr. Aaron Milstone and Dr. Charlotte Woods-Hill at brightstar@jhmi.edu

We are asking you to evaluate 3 metrics about each strategy that you used: its importance, how resource-intensive it was, and how much effort was required to use that strategy.

By “importance,” we mean, how critical was that specific strategy to ultimately reducing blood cultures in your site (for example, did you feel that visual reminders about a new blood culture algorithm were the key way that got your clinicians to change their practices?).

By “resource intensity,” we mean, how much non-human resources (ie, material/monetary/technology resources) were required to use that strategy (example: did you have to spend money to get the IT department to create a new order set in your electronic medical record? Did you have to fund a data analyst’s time in order to obtain and track blood culture data?).

And by “level of effort,” we mean, how much time, or energy, or personal non-monetary investment was required for a specific strategy? (ex: did you have multiple long discussions with a particularly challenging stakeholder before achieving their buy-in? did you spend a lot of personal time to review completed blood culture checklists?)

For strategies that you did NOT use, please answer a few questions about why you did not choose that strategy in your site.

The data we are collecting from this survey will inform our analysis of the overall Bright STAR blood culture QI program, so your responses will be anonymous at the individual level, but we will identify your responses according to your site.

1. Did you secure **leadership** buy-in from any of the following groups for Bright STAR? (excluding team leads) Select all that apply.

- PICU physician leadership, such as division chief, medical director, etc (1)
- PICU nursing leadership (2)
- Frontline clinician leadership, such as fellowship program director, residency program director, or director of nurse practitioner/hospitalist group (3)
- Director of quality/safety or quality/safety committee (4)
- None (5)
- Other - Please describe (6) __________________________________________________

2. How did you engage with leaders about Bright STAR in your site? Select all that apply

- Formal session, such as presentation about Bright STAR to divisional faculty meeting or research meeting; one time (1)
- Formal session, more than once (2)
- Informal discussion in person, one time (3)
- Informal discussion in person, more than once (4)
- Discussion by email, either one time or more than once (5)
- I did not need to engage with anyone to secure leadership buy-in (6)
- Other - Please describe (7) __________________________________________________

3. In general, how important was securing leadership buy-in for achieving blood culture reduction in your site?

- Extremely important (1)
- Very important (2)
- Moderately important (3)
- Slightly important (4)
- Not important at all (5)
- Unsure (6)

4. In general, how resource-intensive was securing leadership buy-in in your site?

- Extremely resource intensive (1)
- Very resource intensive (5)
- Moderately resource intensive (2)
- Slightly resource intensive (3)
- Not resource intensive at all (4)
- Unsure (6)

5. In general, how much effort was required for securing leadership buy-in in your site?

- An extreme degree of effort (1)
- A very high degree of effort (5)
- A moderate degree of effort (2)
- A slight degree of effort (3)
- No effort (I did not do this task) (4)
- Unsure (6)

Task: Secure buy-in of clinical stakeholders

6. Did you engage key stakeholders for **clinical content/clinical buy-in** for Bright STAR? (Select all that apply)

- Oncology (1)
- Stem Cell Transplant (2)
- Solid organ transplant team (such as heart, liver, kidney, etc) (3)
- Nephrology (4)
- Cardiology (5)
- Pulmonary (6)
- Infectious disease (separately from the ID site lead that was part of Bright STAR) (7)
- Advanced practice providers, such as PICU nurse practitioners or hospitalists (8)
- Trainee physicians, such as fellows and residents (9)
- Attending ICU physicians (10)
- PICU bedside nurses (11)
- Phlebotomy or vascular access team (12)
- None (13)
- Other (14)

7. How did you engage with these clinical stakeholders about Bright STAR in your site? Select all that apply

- Formal session, such as presentation about Bright STAR to divisional faculty meeting or research meeting; one time (1)
- Formal session, more than once (2)
- Informal discussion in person, one time (3)
- Informal discussion in person, more than once (4)
- Discussion by email, either one time or more than once (5)
- I did not need to engage with stakeholders (6)
- Other: please describe (7) __________________________________________________

8. In general, how important was clinical stakeholder engagement for achieving blood culture reduction in your site?

- Extremely important (1)
- Very important (5)
- Moderately important (2)
- Slightly important (3)
- Not important at all (4)
- Unsure (6)

9. In general, how resource-intensive was clinical stakeholder engagement in your site?

- Extremely resource intensive (1)
- Very resource intensive (5)
- Moderately resource intensive (2)
- Slightly resource intensive (3)
- Not resource intensive at all (4)
- Unsure (6)

10. In general, how much effort was required for clinical stakeholder engagement in your site?

- An extreme degree of effort (1)
- A very high degree of effort (5)
- A moderate degree of effort (2)
- A slight degree of effort (3)
- No effort (I did not do this task) (4)
- Unsure (6)

Task: Assess context

11. Other than the Bright STAR team-guided Work System Assessment, how did you assess context, such as current blood culture practices and which practices to target for change, during Bright STAR?

- Nothing; we only participated in the study team-guided Work System Assessment (1)
- Something in addition to the WSA, such as internal review or audit of your site’s culture practices, or culture results, etc (Describe more if relevant) (2) _____________________________

12. In general, how important was assessment of pre-project context (ie, the WSA or whatever else your site did) for achieving blood culture reduction in your site?

- Extremely important (1)
- Very important (5)
- Moderately important (2)
- Slightly important (3)
- Not important at all (4)
- Unsure (6)

13. In general, how resource-intensive was assessment of pre-project context (ie, the WSA or whatever else your site did) in your site?

- Extremely resource intensive (1)
- Very resource intensive (5)
- Moderately resource intensive (2)
- Slightly resource intensive (3)
- Not resource intensive at all (4)
- Unsure (6)

14. In general, how much effort was required for assessing pre-project context (ie, the WSA or whatever else your site did) in your site?

- An extreme degree of effort (1)
- A very high degree of effort (5)
- A moderate degree of effort (2)
- A slightly degree of effort (3)
- No effort (I did not do this task) (4)
- Unsure (6)

Task: Establish goals and objectives

15. Did you set a target, such as “10% reduction in total blood culture rate in 6 months” or “decrease use of surveillance cultures on ECMO patients”? Yes/No. If yes, describe any target(s) you set

- Yes (1) __________________________________________________

If yes: establish goals and objectives

16. How important/resource-intensive/effortful was it to **set a target for change**?

|  | Extremely (1) | Very (2) | Moderately (3) | Slightly (4) | Not at all (5) | Unsure (6) |
| --- | --- | --- | --- | --- | --- | --- |
| Importance (1) |  |  |  |  |  |  |
| Resource-Intensive (2) |  |  |  |  |  |  |
| Effort (3) |  |  |  |  |  |  |

- No (2)

If no: establish goals and objectives

16. Which of the following was the reason why you did NOT **set a target**?

- Thought it would be unimportant to change practice at my site (1)
- Thought it would be too resource-intensive (organizational costs) at my site (2)
- Thought it would require too much effort (time, energy) at my site (3)
- Was not considered (i.e. I just didn't think about it) (4)
- Not achievable/feasible at my site (i.e. Not possible at my site) (6)
- Other - Please describe (5) _________________________________________________

Site specific blood culture practices

What type of blood culture tool did your site develop? (Yes/No for each)

17. Paper checklist, requiring completion by clinician and/or collected for review

- Yes (1)

If yes:

18. How important/resource-intensive/effortful was it to have a **paper checklist, requiring completion by clinician and/or collected for review**

|  | Extremely (1) | Very (2) | Moderately (3) | Slightly (4) | Not at all (5) | Unsure (6) |
| --- | --- | --- | --- | --- | --- | --- |
| Importance (1) |  |  |  |  |  |  |
| Resource-Intensive (2) |  |  |  |  |  |  |
| Effort (3) |  |  |  |  |  |  |

- No (2)

If no:

18. Which of the following was the reason why you did NOT develop a **paper checklist, requiring completion by clinician and/or collected for review**? (select ALL that apply)

- Thought it would be unimportant to change practice at my site (1)
- Thought it would be too resource-intensive (organizational costs) at my site (2)
- Thought it would require too much effort (time, energy) at my site (3)
- Was not considered (i.e. I just didn't think about it) (4)
- Not achievable/feasible at my site (i.e. Not possible at my site) (6)
- Other - Please describe (5) _________________________________________________

19. Paper checklist, but not requiring completion and/or not collected for review

- Yes (1)

**If yes:**

20. How important/resource-intensive/effortful was it to have **a paper checklist, but not requiring completion and/or not collected for review?**

|  | Extremely (1) | Very (2) | Moderately (3) | Slightly (4) | Not at all (5) | Unsure (6) |
| --- | --- | --- | --- | --- | --- | --- |
| Importance (1) |  |  |  |  |  |  |
| Resource-Intensive (2) |  |  |  |  |  |  |
| Effort (3) |  |  |  |  |  |  |

- No (2)

If no:

20. Which of the following was the reason why you did NOT develop **a paper checklist, but not requiring completion and/or not collected for review**? (select ALL that apply)

- Thought it would be unimportant to change practice at my site (1)
- Thought it would be too resource-intensive (organizational costs) at my site (2)
- Thought it would require too much effort (time, energy) at my site (3)
- Was not considered (i.e. I just didn't think about it) (4)
- Not achievable/feasible at my site (i.e. Not possible at my site) (6)
- Other - Please describe (5) _________________________________________________

Paper algorithm or flow diagram

21. Paper algorithm or flow diagram

- Yes (1)

If yes:

22. How important/resource-intensive/effortful was it to have **a paper algorithm or flow diagram**?

|  | Extremely (1) | Very (2) | Moderately (3) | Slightly (4) | Not at all (5) | Unsure (6) |
| --- | --- | --- | --- | --- | --- | --- |
| Importance (1) |  |  |  |  |  |  |
| Resource-Intensive (2) |  |  |  |  |  |  |
| Effort (3) |  |  |  |  |  |  |

- No (2)

If no:

22. Which of the following was the reason why you did NOT develop a paper algorithm or flow diagram? (select ALL that apply)

- Thought it would be unimportant to change practice at my site (1)
- Thought it would be too resource-intensive (organizational costs) at my site (2)
- Thought it would require too much effort (time, energy) at my site (3)
- Was not considered (i.e. I just didn't think about it) (4)
- Not achievable/feasible at my site (i.e. Not possible at my site) (6)
- Other - Please describe (5) _________________________________________________

Electronic-health record based tool

23. Electronic-health record based tool, such as pathway or practice guideline

- Yes (1)

If yes:

24. How important/resource-intensive/effortful was it to have **an electronic-health record based tool, such as pathway or practice guideline**?

|  | Extremely (1) | Very (2) | Moderately (3) | Slightly (4) | Not at all (5) | Unsure (6) |
| --- | --- | --- | --- | --- | --- | --- |
| Importance (1) |  |  |  |  |  |  |
| Resource-Intensive (2) |  |  |  |  |  |  |
| Effort (3) |  |  |  |  |  |  |

- No (2)

If no:

24. Which of the following was the reason why you did NOT develop **an electronic-health record based tool**? (select ALL that apply)

- Thought it would be unimportant to change practice at my site (1)
- Thought it would be too resource-intensive (organizational costs) at my site (2)
- Thought it would require too much effort (time, energy) at my site (3)
- Was not considered (i.e. I just didn't think about it) (4)
- Not achievable/feasible at my site (i.e. Not possible at my site) (6)
- Other - Please describe (5) ________________________________________________

25. Other blood culture tools you developed that have not previously been mentioned– please describe

- Yes (1) __________________________________________________

If yes:

26. How important/resource-intensive/effortful was it to have this **other blood culture tool you described**?

|  | Extremely (1) | Very (2) | Moderately (3) | Slightly (4) | Not at all (5) | Unsure (6) |
| --- | --- | --- | --- | --- | --- | --- |
| Importance (1) |  |  |  |  |  |  |
| Resource-Intensive (2) |  |  |  |  |  |  |
| Effort (3) |  |  |  |  |  |  |

- No (2)

Education

27. How did you educate your PICU clinicians about the clinical content in the tool/tools you used? (Yes/No for each)

Formal education session with PICU clinicians &#x1F6C8

- Yes (1)

If yes:

28. How often did the formal education session occur?

- Once (1)
- More than once (2)
- Unsure/Don't Remember (4)

29. How important/resource-intensive/effortful was it to conduct a **formal education session**?

|  | Extremely (1) | Very (2) | Moderately (3) | Slightly (4) | Not at all (5) | Unsure (6) |
| --- | --- | --- | --- | --- | --- | --- |
| Importance (1) |  |  |  |  |  |  |
| Resource-Intensive (2) |  |  |  |  |  |  |
| Effort (3) |  |  |  |  |  |  |

- No (2)

If no:

28. Which of the following was the reason why you did NOT develop **a formal education session with PICU clinicians**? (select ALL that apply)

- Thought it would be unimportant to change practice at my site (1)
- Thought it would be too resource-intensive (organizational costs) at my site (2)
- Thought it would require too much effort (time, energy) at my site (3)
- Was not considered (i.e. I just didn't think about it) (4)
- Not achievable/feasible at my site (i.e. Not possible at my site) (8)
- Other - Please describe (5) _________________________________________________

30. Informal education with PICU clinicians 🛈

- Yes (1)

If yes:

31. How often did the informal education session occur?

- Once (1)
- More than once (2)
- Unsure/Don't remember (4)

32. How important/resource-intensive/effortful was it to have an **informal education with PICU clinicians**?

|  | Extremely (1) | Very (2) | Moderately (3) | Slightly (4) | Not at all (5) | Unsure (6) |
| --- | --- | --- | --- | --- | --- | --- |
| Importance (1) |  |  |  |  |  |  |
| Resource-Intensive (2) |  |  |  |  |  |  |
| Effort (3) |  |  |  |  |  |  |

- No (2)

If no:

31. Which of the following was the reason why you did NOT use **informal education with PICU clinicians**? (select ALL that apply)

- Thought it would be unimportant to change practice at my site (1)
- Thought it would be too resource-intensive (organizational costs) at my site (2)
- Thought it would require too much effort (time, energy) at my site (3)
- Was not considered (i.e. I just didn't think about it) (4)
- Not achievable/feasible at my site (i.e. Not possible at my site) (6)
- Other - Please describe (5) ________________________________________________

33. Visual reminders, such as flyer or screen saver posted in the unit or breakroom for education

- Yes (1)

If yes:

34. How important/resource-intensive/effortful was it to use **a flyer or screen saver posted in the unit or breakroom for education**?

|  | Extremely (1) | Very (2) | Moderately (3) | Slightly (4) | Not at all (5) | Unsure (6) |
| --- | --- | --- | --- | --- | --- | --- |
| Importance (1) |  |  |  |  |  |  |
| Resource-Intensive (2) |  |  |  |  |  |  |
| Effort (3) |  |  |  |  |  |  |

- No (2)

If no:

34. Which of the following was the reason why you did NOT use **visual reminders, such as flyer or screen saver posted in the unit or breakroom for education**? (select ALL that apply)

- Thought it would be unimportant to change practice at my site (1)
- Thought it would be too resource-intensive(organizational costs) at my site (2)
- Thought it would require too much effort (time, energy) at my site (3)
- Was not considered (i.e. I just didn't think about it) (4)
- Not achievable/feasible at my site (i.e. Not possible at my site) (6)
- Other - Please describe (5) _________________________________________________

35. Email announcement for education

- Yes (1) \

If yes:

36. How often did you send an email announcement for education?

- Once (1)
- More than once (2)
- Unsure/Don't remember (4)

37. How important/resource-intensive/effortful was it to send **email announcement(s) for education**?

|  | Extremely (1) | Very (2) | Moderately (3) | Slightly (4) | Not at all (5) | Unsure (6) |
| --- | --- | --- | --- | --- | --- | --- |
| Importance (1) |  |  |  |  |  |  |
| Resource-Intensive (2) |  |  |  |  |  |  |
| Effort (3) |  |  |  |  |  |  |

- No (2)

If no:

36. Which of the following was the reason why you did NOT use **an email announcement for education**? (select ALL that apply)

- Thought it would be unimportant to change practice at my site (1)
- Thought it would be too resource-intensive (organizational costs) at my site (2)
- Thought it would require too much effort (time, energy) at my site (3)
- Was not considered (i.e. I just didn't think about it) (4)
- Not achievable/feasible at my site (i.e. Not possible at my site) (6)
- Other - Please describe (5) _________________________________________________

38. Online education module

- Yes (1)

If yes:

39. How important/resource-intensive/effortful was it to use an **online education module**?

|  | Extremely (1) | Very (2) | Moderately (3) | Slightly (4) | Not at all (5) | Unsure (6) |
| --- | --- | --- | --- | --- | --- | --- |
| Importance (1) |  |  |  |  |  |  |
| Resource-Intensive (2) |  |  |  |  |  |  |
| Effort (3) |  |  |  |  |  |  |

- No (2)

If no:

39. Which of the following was the reason why you did NOT use **an online education module**? (select ALL that apply)

- Thought it would be unimportant to change practice at my site (1)
- Thought it would be too resource-intensive (organizational costs) at my site (2)
- Thought it would require too much effort (time, energy) at my site (3)
- Was not considered (i.e. I just didn't think about it) (4)
- Not achievable/feasible at my site (i.e. Not possible at my site) (6)
- Other - Please describe (5) _________________________________________________

40. Other education tools you developed that were not previously mentioned – please describe

- Yes (1) __________________________________________________

If yes:

41. How important/resource-intensive/effortful was it use this **other type of education you described**?

|  | Extremely (1) | Very (2) | Moderately (3) | Slightly (4) | Not at all (5) | Unsure (6) |
| --- | --- | --- | --- | --- | --- | --- |
| Importance (1) |  |  |  |  |  |  |
| Resource-Intensive (2) |  |  |  |  |  |  |
| Effort (3) |  |  |  |  |  |  |

- No (2)

Advertising

42. Formal session to advertise/make PICU clinicians aware of this clinical tool (ex: at regular staff meeting, or unit-wide conference or educational session)

- Yes (1)

If yes:

43. How often did the formal session to advertise/make PICU clinicians aware of the clinical tool occur?

- Once (1)
- More than once (2)
- Unsure/Don't remember (4)

44. How important/resource-intensive/effortful was it to conduct **a formal session to advertise/make PICU clinicians aware of this clinical tool (ex: at regular staff meeting, or unit-wide conference or educational session)**?

|  | Extremely (1) | Very (2) | Moderately (3) | Slightly (4) | Not at all (5) | Unsure (6) |
| --- | --- | --- | --- | --- | --- | --- |
| Importance (1) |  |  |  |  |  |  |
| Resource-Intensive (2) |  |  |  |  |  |  |
| Effort (3) |  |  |  |  |  |  |

- No (2)

If no:

43. Which of the following was the reason why you did NOT use **a formal session to advertise/make PICU clinicians aware of this clinical tool (ex: at regular staff meeting, or unit-wide conference or educational session)?**

- Thought it would be unimportant to change practice at my site (1)
- Thought it would be too resource-intensive (organizational costs) at my site (2)
- Thought it would require too much effort (time, energy) at my site (3)
- Was not considered (i.e. I just didn't think about it) (4)
- Not achievable/feasible at my site (i.e. Not possible at my site) (6)
- Other - Please describe (5) _________________________________________________

45. Informal discussion to advertise/make PICU clinicians aware of the clinical tool (ex: describing it during team rounds or when on-call in the unit)

- Yes (1)

If yes:

46. How often did this informal discussion to advertise/make PICU clinicians aware of this clinical tool occur?

- Once (1)
- More than once (2)
- Unsure/Don't remember (4)

47. How important/resource-intensive/effortful was it to conduct **an informal discussion to advertise/make PICU clinicians aware of this clinical tool (ex: describing it during team rounds or when on-call in the unit)**?

|  | Extremely (1) | Very (2) | Moderately (3) | Slightly (4) | Not at all (5) | Unsure (6) |
| --- | --- | --- | --- | --- | --- | --- |
| Importance (1) |  |  |  |  |  |  |
| Resource-Intensive (2) |  |  |  |  |  |  |
| Effort (3) |  |  |  |  |  |  |

- No (2)

If no:

46. Which of the following was the reason why you did NOT use **an informal discussion to advertise/make PICU clinicians aware of this clinical tool (ex: describing it during team rounds or when on-call in the unit)**?

- Thought it would be unimportant to change practice at my site (1)
- Thought it would be too resource-intensive (organizational costs) at my site (2)
- Thought it would require too much effort (time, energy) at my site (3)
- Was not considered (i.e. I just didn't think about it) (5)
- Not achievable/feasible at my site (i.e. Not possible at my site) (6)
- Other - Please describe (4) _________________________________________________

48. Visual reminders, such as flyer or screen saver posted in the unit or breakroom to advertise/make PICU clinicians aware of this clinical tool

- Yes (1)

If yes:

49. How important/resource-intensive/effortful was it to use **visual reminders, such as flyer or screen saver posted in the unit or breakroom for advertise/make PICU clinicians aware of this clinical tool?**

|  | Extremely (1) | Very (2) | Moderately (3) | Slightly (4) | Not at all (5) | Unsure (6) |
| --- | --- | --- | --- | --- | --- | --- |
| Importance (1) |  |  |  |  |  |  |
| Resource-Intensive (2) |  |  |  |  |  |  |
| Effort (3) |  |  |  |  |  |  |

- No (2)

If no:

49. Which of the following was the reason why you did NOT use **visual reminders, such as flyer or screen saver posted in the unit or breakroom to advertise/make PICU clinicians aware of this clinical tool**?

- Thought it would be unimportant to change practice at my site (1)
- Thought it would be too resource-intensive (organizational costs) at my site (2)
- Thought it would require too much effort (time, energy) at my site (3)
- Was not considered (i.e. I just didn't think about it) (4)
- Not achievable/feasible at my site (i.e. Not possible at my site) (6)
- Other - Please describe (5) _________________________________________________

50. Email(s) to advertise/make PICU clinicians aware of this clinical tool

- Yes (1)

If yes:

51. How often did you send an email to advertise/make PICU clinicians aware of this clinical tool?

- Once (1)
- More than once (2)
- Unsure/Don't remember (4)

52. How important/resource-intensive/effortful was it to send **email(s) to advertise/make PICU clinicians aware of this clinical tool**?

|  | Extremely (1) | Very (2) | Moderately (3) | Slightly (4) | Not at all (5) | Unsure (6) |
| --- | --- | --- | --- | --- | --- | --- |
| Importance (1) |  |  |  |  |  |  |
| Resource-Intensive (2) |  |  |  |  |  |  |
| Effort (3) |  |  |  |  |  |  |

- No (2)

If no:

51. Which of the following was the reason why you did NOT use **email(s) to advertise/make PICU clinicians aware of this clinical tool**? (select ALL that apply)

- Thought it would be unimportant to change practice at my site (1)
- Thought it would be too resource-intensive (organizational costs) at my site (2)
- Thought it would require too much effort (time, energy) at my site (3)
- Was not considered (i.e. I just didn't think about it) (4)
- Not achievable/feasible at my site (i.e. Not possible at my site) (6)
- Other - Please describe (5) _________________________________________________

53. Financial incentives (such as money or gift card for completing a checklist) to advertise/make PICU clinicians aware of this clinical tool

- Yes (1)

If yes:

54. How important/resource-intensive/effortful was it to use **financial incentives (such as money or gift card for completing a checklist) to advertise/make PICU clinicians aware of this clinical tool?**

|  | Extremely (1) | Very (2) | Moderately (3) | Slightly (4) | Not at all (5) | Unsure (6) |
| --- | --- | --- | --- | --- | --- | --- |
| Importance (1) |  |  |  |  |  |  |
| Resource-Intensive (2) |  |  |  |  |  |  |
| Effort (3) |  |  |  |  |  |  |

- No (2)

If no:

54. Which of the following was the reason why you did NOT use **financial incentives (such as money or gift card for completing a checklist) to advertise/make PICU clinicians aware of this clinical tool**?

- Thought it would be unimportant to change practice at my site (1)
- Thought it would be too resource-intensive (organizational costs) at my site (2)
- Thought it would require too much effort (time, energy) at my site (3)
- Was not considered (i.e. I just didn't think about it) (4)
- Not achievable/feasible at my site (i.e. Not possible at my site) (6)
- Other - Please describe (5) _________________________________________________

55. Other incentives (such as a “shout out” or being named “star of the shift” for adhering to the blood culture tool) to advertise/make PICU clinicians aware of this clinical tool?

- Yes (1)

If yes:

56. How important/resource-intensive/effortful was it to use these other **original incentives (such as a “shout out” or being named “star of the shift” for adhering to the blood culture tool) to advertise/make PICU clinicians aware of this clinical tool?**

|  | Extremely (1) | Very (2) | Moderately (3) | Slightly (4) | Not at all (5) | Unsure (6) |
| --- | --- | --- | --- | --- | --- | --- |
| Importance (1) |  |  |  |  |  |  |
| Resource-Intensive (2) |  |  |  |  |  |  |
| Effort (3) |  |  |  |  |  |  |

- No (2)

If no:

56. Which of the following was the reason why you did NOT use **other incentives (such as a “shout out” or being named “star of the shift” for adhering to the blood culture tool) to advertise/make PICU clinicians aware of this clinical tool**? (select ALL that apply)

- Thought it would be unimportant to change practice at my site (1)
- Thought it would be too resource-intensive (organizational costs) at my site (2)
- Thought it would require too much effort (time, energy) at my site (3)
- Was not considered (i.e. I just didn't think about it) (4)
- Not achievable/feasible at my site (i.e. Not possible at my site) (6)
- Other - Please describe (5) _________________________________________________

57. Other forms of advertisement/awareness of this clinical tool with PICU clinicians you developed that were not previously mentioned– please describe

- Yes (1) __________________________________________________

If yes:

58. How important/resource-intensive/effortful was this **other form of advertisement/awareness of this clinical tool with PICU clinicians you described?**

|  | Extremely (1) | Very (2) | Moderately (3) | Slightly (4) | Not at all (5) | Unsure (6) |
| --- | --- | --- | --- | --- | --- | --- |
| Importance (1) |  |  |  |  |  |  |
| Resource-Intensive (2) |  |  |  |  |  |  |
| Effort (3) |  |  |  |  |  |  |

- No (2)

Workflow Changes

59. Integration of new blood culture practices into sepsis huddle

- Yes (1)

If yes:

60. How important/resource-intensive/effortful was **integration of new blood culture practices into sepsis huddle**?

|  | Extremely (1) | Very (2) | Moderately (3) | Slightly (4) | Not at all (5) | Unsure (6) |
| --- | --- | --- | --- | --- | --- | --- |
| Importance (1) |  |  |  |  |  |  |
| Resource-Intensive (2) |  |  |  |  |  |  |
| Effort (3) |  |  |  |  |  |  |

- No (2)

If no:

60. Which of the following was the reason why you did NOT use **integration of new blood culture practices into sepsis huddle**? (select ALL that apply)

- Thought it would be unimportant to change practice at my site (1)
- Thought it would be too resource-intensive (organizational costs) at my site (2)
- Thought it would require too much effort (time, energy) at my site (3)
- Was not considered (i.e. I just didn't think about it) (4)
- Not achievable/feasible at my site (i.e. Not possible at my site) (6)
- Other - Please describe (5) _________________________________________________

61. Integration of a physical exam before blood culture order decision is made

- Yes (1)

If yes:

62. How important/resource-intensive/effortful was **integration of a physical exam before blood culture order decision is made**?

|  | Extremely (1) | Very (2) | Moderately (3) | Slightly (4) | Not at all (5) | Unsure (6) |
| --- | --- | --- | --- | --- | --- | --- |
| Importance (1) |  |  |  |  |  |  |
| Resource-Intensive (2) |  |  |  |  |  |  |
| Effort (3) |  |  |  |  |  |  |

- No (2)

If no:

62. Which of the following was the reason why you did NOT use **integration of a physical exam before blood culture order decision is made**? (select ALL that apply)

- Thought it would be unimportant to change practice at my site (1)
- Thought it would be too resource-intensive (organizational costs) at my site (2)
- Thought it would require too much effort (time, energy) at my site (3)
- Was not considered (i.e. I just didn't think about it) (4)
- Not achievable/feasible at my site (i.e. Not possible at my site) (6)
- Other - Please describe (5) _________________________________________________

63. Attempt for a peripheral blood culture instead of or in addition to a central venous catheter blood culture

- Yes (1)

If yes:

64. How important/resource-intensive/effortful was **an attempt for a peripheral blood culture instead of or in addition to a central venous catheter blood culture**?

|  | Extremely (1) | Very (2) | Moderately (3) | Slightly (4) | Not at all (5) | Unsure (6) |
| --- | --- | --- | --- | --- | --- | --- |
| Importance (1) |  |  |  |  |  |  |
| Resource-Intensive (2) |  |  |  |  |  |  |
| Effort (3) |  |  |  |  |  |  |

- No (2)

If no:

64. Which of the following was the reason why you did NOT use **an attempt for a peripheral blood culture instead of or in addition to a central venous catheter blood culture**? (select ALL that apply)

- Thought it would be unimportant to change practice at my site (1)
- Thought it would be too resource-intensive (organizational costs) at my site (2)
- Thought it would require too much effort (time, energy) at my site (3)
- Was not considered (i.e. I just didn't think about it) (4)
- Not achievable/feasible at my site (i.e. Not possible at my site) (6)
- Other - Please describe (5) _________________________________________________

65. Creation of new blood culture orderset in electronic health record, or edits to existing orderset

- Yes (1)

If yes:

66. How important/resource-intensive/effortful was the **creation of a new blood culture orderset in electronic health record, or edits to existing orderset?**

|  | Extremely (1) | Very (2) | Moderately (3) | Slightly (4) | Not at all (5) | Unsure (6) |
| --- | --- | --- | --- | --- | --- | --- |
| Importance (1) |  |  |  |  |  |  |
| Resource-Intensive (2) |  |  |  |  |  |  |
| Effort (3) |  |  |  |  |  |  |

- No (2)

If no:

66. Which of the following was the reason why you did NOT use **creation of new blood culture orderset in electronic health record, or edits to existing orderset**? (select ALL that apply)

- Thought it would be unimportant to change practice at my site (1)
- Thought it would be too resource-intensive (organizational costs) at my site (2)
- Thought it would require too much effort (time, energy) at my site (3)
- Was not considered (i.e. I just didn't think about it) (4)
- Not achievable/feasible at my site (i.e. Not possible at my site) (6)
- Other - Please describe (5) _________________________________________________

67. Designation or new team or personnel to physically collect blood cultures (ex: a dedicated phlebotomy team, or responsibility of PICU clinician to now collect, etc)

- Yes (1)

If yes:

68. How important/resource-intensive/effortful was **designation or new team or personnel to physically collect blood cultures (ex: a dedicated phlebotomy team, or responsibility of PICU clinician to now collect, etc)?**

|  | Extremely (1) | Very (2) | Moderately (3) | Slightly (4) | Not at all (5) | Unsure (6) |
| --- | --- | --- | --- | --- | --- | --- |
| Importance (1) |  |  |  |  |  |  |
| Resource Intensive (2) |  |  |  |  |  |  |
| Effort (3) |  |  |  |  |  |  |

- No (2)

If no:

68. Which of the following was the reason why you did NOT use **designation or new team or personnel to physically collect blood cultures (ex: a dedicated phlebotomy team, or responsibility of PICU clinician to now collect, etc)**? (select ALL that apply)

- Thought it would be unimportant to change practice at my site (1)
- Thought it would be too resource-intensive (organizational costs) at my site (2)
- Thought it would require too much effort (time, energy) at my site (3)
- Was not considered (i.e. I just didn't think about it) (4)
- Not achievable/feasible at my site (i.e. Not possible at my site) (6)
- Other - Please describe (5) _________________________________________________

69. Change in sample collection sequence for blood cultures, such as decision to attempt to obtain peripheral specimen before a central venous catheter sample

- Yes (1)

If yes:

70. How important/resource-intensive/effortful was **a change in sample collection sequence for blood cultures, such as decision to attempt to obtain peripheral specimen before a central venous catheter sample?**

|  | Extremely (1) | Very (2) | Moderately (3) | Slightly (4) | Not at all (5) | Unsure (6) |
| --- | --- | --- | --- | --- | --- | --- |
| Importance (1) |  |  |  |  |  |  |
| Resource-Intensive (2) |  |  |  |  |  |  |
| Effort (3) |  |  |  |  |  |  |

- No (2)

If no:

70. Which of the following was the reason why you did NOT use **change in sample collection sequence for blood cultures, such as decision to attempt to obtain peripheral specimen before a central venous catheter sample**? (select ALL that apply)

- Thought it would be unimportant to change practice at my site (1)
- Thought it would be too resource-intensive (organizational costs) at my site (2)
- Thought it would require too much effort (time, energy) at my site (3)
- Was not considered (i.e. I just didn't think about it) (4)
- Not achievable/feasible at my site (i.e. Not possible at my site) (6)
- Other - Please describe (5) _________________________________________________

71. Other types of workflow changes you implemented that were not previously mentioned – please describe

- Yes (1) __________________________________________________

If yes:

72. How important/resource-intensive/effortful was this **other type of workflow change you implemented**?

|  | Extremely (1) | Very (2) | Moderately (3) | Slightly (4) | Not at all (5) | Unsure (6) |
| --- | --- | --- | --- | --- | --- | --- |
| Importance (1) |  |  |  |  |  |  |
| Resource-Intensive (2) |  |  |  |  |  |  |
| Effort (3) |  |  |  |  |  |  |

- No (2)

Adapt blood culture tool

73. How did you revise/adapt your site’s blood culture tool after launching the initial version? Select one

- Edits to the paper tool, one time (1)
- Edits to the paper tool, more than once (How many revisions? Please describe) (2) __________________________________________________
- Edits to the EHR-based or online tool, one time (3)
- Edits to the EHR-based or online tool, more than once (How many revisions? Please describe) (4) __________________________________________________
- We never made any changes to the tool after introducing it at my site (5)
- Other – please describe (6) __________________________________________________

If yes:

What generally prompted these revisions/adaptions to your site’s blood culture tool? (Yes; No; Unsure/Neutral for each)

74. Blood cultures were not decreasing as much as we hoped

- Yes (Did the revisions address the issue? Please describe) (1) ___________________
- No (2)
- Unsure/Neutral (3)

75. Blood cultures were decreasing too much

- Yes (Did the revisions address the issue? Please describe) (1)_____________________
- No (2)
- Unsure/Neutral (3)

76. PICU clinician/stakeholder feedback about the tool

- Yes (Did the revisions address the issue? Please describe) (1) ____________________
- No (2)
- Unsure/Neutral (3)

77. Non-PICU stakeholder feedback about the tool (such as comments from Oncology, Phlebotomy team, etc)

- Yes (Did the revisions address the issue? Please describe) (1) _____________________
- No (2)
- Unsure/Neutral (3)

78. Other feedback/occurrences not previously mentioned that prompted you to revise your tool - Please describe

- Yes (Did the revisions address the issue? Please describe) (1) ____________________
- No (2)
- Unsure/Neutral (3)

79. Did you/how did you revise your strategies (ie, what you selected for blood culture tools, education, advertisement, etc) after the initial launch of the project in your site? For example, did you make different flyers/posters; stop requiring a signed checklist; or stop doing financial incentives?___________________________________________________

Evaluate processes and outcomes: Shared compliance data

Early in Bright STAR, you were asked to audit cultures to see how many were compliant with your site’s new blood culture approach.

80. How important/resource-intensive/effortful was collecting the compliance data to share **with the main Bright STAR study team**?

|  | Extremely (1) | Very (2) | Moderately (3) | Slightly (4) | Not at all (5) | Unsure (6) |
| --- | --- | --- | --- | --- | --- | --- |
| Importance (1) |  |  |  |  |  |  |
| Resource-Intensive (2) |  |  |  |  |  |  |
| Effort (3) |  |  |  |  |  |  |

81. Did you share compliance data with clinicians/stakeholders at your site?

- Yes (1)

If yes:

82. How often did you share compliance data with clinicians/stakeholders at your site?

- Once (1)
- More than once (2)
- Unsure/Don't remember (4)
- No (2)

If no:

82. Which of the following was the reason why you did NOT **share compliance data with clinicians/stakeholders at your site**(select ALL that apply)?

- Thought it would be unimportant to change practice at my site (1)
- Thought it would be too resource-intensive (organizational costs) at my site (2)
- Thought it would require too much effort (time, energy) at my site (3)
- Was not considered (i.e. I just didn't think about it) (4)
- Not achievable/feasible at my site (i.e. Not possible at my site) (6)
- Other - Please describe (5) _________________________________________________

83. How important/resource-intensive/effortful was it to **share compliance data with clinicians/stakeholders at your site**?

|  | Extremely (1) | Very (2) | Moderately (3) | Slightly (4) | Not at all (5) | Unsure (6) |
| --- | --- | --- | --- | --- | --- | --- |
| Importance (1) |  |  |  |  |  |  |
| Resource-Intensive (2) |  |  |  |  |  |  |
| Effort (3) |  |  |  |  |  |  |

Sharing data

84. How often did you share rate data as feedback for the following groups?

|  | Steadily throughout project (2) | Increasing frequency as study progressed (3) | Decreasing frequency as study progressed (4) | Did not share data with this group (5) |
| --- | --- | --- | --- | --- |
| Main Bright STAR JHU Collaborative Team (1) |  |  |  |  |
| Internal Project Team at Site (2) |  |  |  |  |
| Group of Clinicians/Stakeholders at Site (4) |  |  |  |  |
| Individual Clinicians/Stakeholders at Site (5) |  |  |  |  |

85. During Bright STAR, the study team shared data about all sites’ performance periodically during collaborative calls. Did you use that data for performance benchmarking during Bright STAR at your site? (Yes/No for each)

Shared data about my site’s results vs other Bright STAR sites’ results with members of my internal project team

- Yes (1)

If yes:

86. How important/resource-intensive/effortful was it to **share data about your site’s results vs other Bright STAR sites’ results with members of your internal project team?**

|  | Extremely (1) | Very (2) | Moderately (3) | Slightly (4) | Not at all (5) | Unsure (6) |
| --- | --- | --- | --- | --- | --- | --- |
| Importance (1) |  |  |  |  |  |  |
| Resource-Intensive (2) |  |  |  |  |  |  |
| Effort (3) |  |  |  |  |  |  |

- No (2)

If no:

86. Which of the following was the reason why you did NOT **share data about my site’s results vs other Bright STAR sites’ results with members of my internal project team**? (select ALL that apply)

- Thought it would be unimportant to change practice at my site (1)
- Thought it would be too resource-intensive (organizational costs) at my site (2)
- Thought it would require too much effort (time, energy) at my site (3)
- Was not considered (i.e. I just didn't think about it) (4)
- Not achievable/feasible at my site (i.e. Not possible at my site) (6)
- Other - Please describe (5) ________________________________________________

87. Shared data about my site’s results vs other Bright STAR sites’ results with members of my PICU/institution such as key stakeholders

- Yes (1)

If yes:

88. How important/resource-intensive/effortful was it to **share your site’s results vs other Bright STAR sites’ results with members of your PICU/institution such as key stakeholders**?

|  | Extremely (1) | Very (2) | Moderately (3) | Slightly (4) | Not at all (5) | Unsure (6) |
| --- | --- | --- | --- | --- | --- | --- |
| Importance (1) |  |  |  |  |  |  |
| Resource-Intensive (2) |  |  |  |  |  |  |
| Effort (3) |  |  |  |  |  |  |

- No (2)

If no:

88. Which of the following was the reason why you did NOT **share data about your site’s results vs other Bright STAR sites’ results with members of your PICU/institution such as key stakeholders**? (select ALL that apply)

- Thought it would be unimportant to change practice at my site (1)
- Thought it would be too resource-intensive (organizational costs) at my site (2)
- Thought it would require too much effort (time, energy) at my site (3)
- Was not considered (i.e. I just didn't think about it) (4)
- Not achievable/feasible at my site (i.e. Not possible at my site) (6)
- Other - Please describe (5) _________________________________________________

89. Did you share data about other sites’ results with **anyone** at your site?

- Yes (1)

If yes:

90. How important/resource-intensive/effortful was it to share data about other sites’ results with **anyone** at your site?

|  | Extremely (1) | Very (2) | Moderately (3) | Slightly (4) | Not at all (5) | Unsure (6) |
| --- | --- | --- | --- | --- | --- | --- |
| Importance (1) |  |  |  |  |  |  |
| Resource-Intensive (2) |  |  |  |  |  |  |
| Effort (3) |  |  |  |  |  |  |

- No (2)

If no:

90. Which of the following was the reason why you did NOT share data about other sites’ results with anyone at your site? (select ALL that apply)

- Thought it would be unimportant to change practice at my site (1)
- Thought it would be too resource-intensive (organizational costs) at my site (2)
- Thought it would require too much effort (time, energy) at my site (3)
- Was not considered (i.e. I just didn't think about it) (4)
- Not achievable/feasible at my site (i.e. Not possible at my site) (6)
- Other - Please describe (5) __________________________________________________

91. Did you use some other process to monitor results and outcomes in your site? Please describe

_______________________________________________________________
