# Supplementary material for: Diagnostic stewardship for blood cultures in the pediatric intensive care unit: lessons in implementation from the BrighT STAR Collaborative
Source: Antimicrob Steward Healthc Epidemiol. 2024 Sep 25;4(1):e148. doi: 10.1017/ash.2024.416 (PMC11428017; doi:10.1017/ash.2024.416)
Supplement: Woods-Hill et al. supplementary material 2 — Woods-Hill et al. supplementary material [file S2732494X24004169sup002.docx]

**Appendix of authors**

Asya Agulnik MD^1^, J Elaine-Marie Albert MD MHA^2^, Michael J Auth DO^3^, Jason A Clayton MD PhD^4^, Susan E Coffin MD MPH^5^, Samantha Dallefeld MD^6^, Chidiebere P Ezetendu MD^7^, Nina A Fainberg MD^8^, Brian F Flaherty MD^9^, Charles B Foster MD^10^, Sarmistha B Hauger MD^11^, Sue J Hong MD^12^, Nicholas D. Hysmith MD^13^, Aileen L Kirby MD FAAP^14^, Larry K Kociolek MD MSCI^15^, Gitte Y Larsen MD MPH^16^, John C Lin MD^17^, William M Linam MD MS^18^, Jason G Newland MD Med^19^, Dawn Nolt MD MPH^20^, Gregory P Priebe MD^21^, Thomas J Sandora MD MPH^22^, Hayden T Schwenk MD MPH^23^, Craig M Smith MD^24^, Katherine M. Steffen MD MHS^25^, Sachin D Tadphale MBBS MPH FAAP^26^, Philp Toltzis MD^27^, Joshua Wolf MBBS, PhD, FRACP^28^, Danielle M Zerr MD MPH^29^

^1^ Department of Global Pediatric Medicine, Division of Critical Care, St Jude Children’s Research Hospital

^2^ Division of Pediatric Critical Care Medicine, Department of Pediatrics, Seattle Children’s Hospital and the University of Washington

^3^ Division of Pediatric Critical Care, Department of Pediatrics, Dell Medical School, University of Texas at Austin

^4^ University Hospitals, Rainbow Babies & Children's Hospital

^5^ Children’s Hospital of Philadelphia, Perelman School of Medicine at University of Pennsylvania

^6^ Division of Pediatric Critical Care, Department of Pediatrics, Dell Medical School, University of Texas at Austin

^7^ Cleveland Clinic Children’s Hospital

^8^ Division of Critical Care Medicine, Children’s Healthcare of Atlanta at Egleston, Department of Pediatrics, Emory University School of Medicine

^9^ University of Utah, Department of Pediatrics, Division of Critical Care

^10^ Section of Pediatric Infectious Diseases, Cleveland Clinic Children’s Hospital

^11^Division of Pediatric Infectious Disease, Dell Medical School, University of Texas at Austin

^12^Divisions of Critical Care and Neurology, Ann & Robert H. Lurie Children’s Hospital of Chicago, Department of Pediatrics, Northwestern University Feinberg School of Medicine

^13^Department of Pediatrics, University of Tennessee Health Science Center, Le Bonheur Children’s Hospital

^14^ Division of Pediatric Critical Care, Doernbecher Children's Hospital/OHSU

^15^ Department of Pediatrics, Northwestern University Feinberg School of Medicine, Ann & Robert H Lurie Children’s Hospital of Chicago

^16^ Division of Critical Care Medicine, Department of Pediatrics, Intermountain Primary Children’s Hospital and University of Utah

^17^ Division of Pediatric Critical Care Medicine, Department of Pediatrics, Washington University School of Medicine

^18^ Emory School of Medicine and Children's Healthcare of Atlanta

^19^ Washington University School of Medicine

^20^ Doernbecher Children's Hospital/Oregon Health and Science University

^21^Division of Critical Care Medicine, Department of Anesthesiology, Critical Care and Pain Medicine, Boston Children’s Hospital; Division of Infectious Diseases, Department of Pediatrics, Boston Children’s Hospital; Department of Anesthesia, Harvard Medical School

^22^ Division of Infectious Diseases, Boston Children’s Hospital; Department of Pediatrics, Harvard Medical School

^23^ Stanford University School of Medicine, Lucile Packard Children’s Hospital Stanford

^24^ Departments of Pediatrics and Neurology, Northwestern University Feinberg School of Medicine,
Ann & Robert H. Lurie Children’s Hospital of Chicago

^25^ Stanford University Department of Pediatrics, Division of Pediatric Critical Care Medicine

^26^ Division of Pediatric Critical Care & Cardiology, Department of Pediatrics, Le Bonheur Children’s Hospital, University of Tennessee Health Science Center

^27^ Rainbow Babies and Children’s Hospital
^28^ Department of Infectious Diseases, St. Jude Children's Research Hospital; Department of Pediatrics, University of Tennessee Health Science Center

^29^ Seattle Children’s Hospital and Department of Pediatrics, University of Washington
